# Comparative Pharmacokinetics of Cinobufacini Capsule and Injection by UPLC-MS/MS

**DOI:** 10.3389/fphar.2022.944041

**Published:** 2022-07-18

**Authors:** Ming Li, Yanhong Qin, Zhe Li, Jinshuai Lan, Tong Zhang, Yue Ding

**Affiliations:** ^1^ Experiment Center of Teaching and Learning, Shanghai University of Traditional Chinese Medicine, Shanghai, China; ^2^ School of Pharmacy, Shanghai University of Traditional Chinese Medicine, Shanghai, China

**Keywords:** comparative pharmacokinetics, cinobufacini capsule, cinobufacini injection, UPLC-MS/MS, bufalin

## Abstract

Cinobufacini capsule and injection are two different formulations from the same source, obtained from the extraction of the skin of *Bufo bufo gargarizans* Cantor, which have been approved by the Chinese State Food and Drug Administration (CFDA) for the treatment of various cancers. Our previous study has found that the cinobufacini capsule and injection exhibited different anticancer effects, but their different pharmacokinetic behaviors, which could give a cause of that, have never been reported. So a sensitive and selective method for the simultaneous quantitation of 13 compounds in the rat plasma, including bufothionine, hellebrigenin, bufalin, gamabufotalin, telocinobufagin, cinobufagin, arenobufagin, cinobufotalin, desacetylcinobufotalin, bufotalin, pseudobufarenogin, resibufogenin, and desacetylcinobufagin, was established by using the Agilent 6460 mass spectrometer equipped with an ESI ion source in a multiple-reaction monitoring (MRM) mode. Chromatographic analysis was accomplished in 6 min by using an Agilent SB-C18 column and a mobile phase consisting of 0.1% formic acid in water and acetonitrile in an optimized gradient program at a flow rate of 0.3 ml/min. The correlation coefficients (r) of all analytes ranged from 0.9967 to 0.9996, while their lower limits of quantification ranged from 0.20 to 4.84 ng/ml. The method has been fully verified and applied for the pharmacokinetic difference study of the Cinobufacini capsule and injection in rats. The results showed that nine components could be quantitated in rat plasma samples after the administration of the cinobufacini capsule, while only bufothionine, bufalin, arenobufagin, and pseudobufarenogin could be detected in the cinobufacini injection group. Their pharmacokinetic studies indicated telocinobufagin, bufalin, desacetylcinobufagin, and arenobufagin were predicted as the potential active substances of the Cinobufacini capsule, while bufothionine was considered as a major ingredient in the cinobufacini injection due to its relatively high blood drug exposure. Also, the AUC of the nine components in cinobufacini capsule groups with three different doses showed a similar trend with significant differences, and the exposure increased with the increase of the dose. The pharmacokinetic characteristics of all major ingredients in cinobufacini capsules and injection were of wide variation, which could be used to explain differences in the efficacy of the cinobufacini capsule and injection and infer the pharmacodynamic ingredients of various cinobufacini preparations.

## 1 Introduction

Cancer is considered as the critical public health problem and the second leading cause of morbidity and mortality worldwide, according to GLOBOCAN 2020 official statistics from the World Health Organization (WHO) in 2020. New cases of cancer have reached 19.3 million, and the number of cancer deaths is up to 10 million in 2020 (Sung et al., 2021). Although various preventative and curative interventions including surgery operation, chemotherapy, and radiation treatment have been used as critical oncological therapeutic strategies, they could not ameliorate their high mortality rates over the last few decades due to the numerous shortcomings of current treatments (Zhong et al., 2021). The chemotherapy combined with surgical operation, radiotherapy, and biotherapy was chosen as the major medical practice for cancer treatment. However, chemotherapy was mainly used to kill tumor cells and inhibit tumor growth and metastasis but sometimes failed to discriminate cancerous cells from normal cells, causing serious toxicity and side effects. Most common chemotherapy drugs, such as sorafenib, regorafenib, and lenvatinib, have been reported to induce side effects, including diarrhea, weight loss, and hair loss (Mansouri et al., 2021). Moreover, low response rate and drug resistance were other challenges for chemotherapy, so it is urgent to develop new effective anti-cancer drugs with low toxicity and making the patients “survival with cancer” for a long time.

Traditional Chinese medicine (TCM) has been widely used to treat cancers in China and other East Asian countries, as adjunctive or complementary therapy. The empirical applications have indicated that TCM not only enhances life quality and progression-free survival in patients with cancer through multi-pathways but also diminishes adverse reactions of chemotherapy, radiotherapy, or targeted therapy (Huang et al., 2020). Some famous herbal formulae have been developed into the Chinese patent medicine and approved by the China Food and Drug Administration (CFDA) for cancer treatment, such as cinobufacini capsule, cinobufacini injection (Xu et al., 2019; Peng et al., 2021), Aidi injection (Yang et al., 2022), Shenyi capsule, Xiaoaiping injection (Huang et al., 2013), and Kanglaite injection (Fu et al., 2014). Cinobufacini is a well-known Chinese medicine, from the boiling water extraction of the skin of *Bufo bufo gargarizans* Cantor, also called Huachansu in Chinese. It has been used clinically for various cancers, including hepatocellular carcinoma (Qi et al., 2018a), colon cancer (Wang et al., 2020), and gastric cancer (Shen et al., 2018). The anticancer mechanisms of cinobufacini mainly included the effects of inducing cancer cell apoptosis, inhibiting cancer cell proliferation, and metastasis (Nakata et al., 2015; Yang et al., 2015; Chen et al., 2018). It has been approved by the FDA in two preparation forms of the cinobufacini capsule and injection for the clinical treatment of cancer. Although the methods for the simultaneous determination of major components including eight bufadienolides and other compounds in Cinobufacini injection have been developed by LC-MS/MS and HPLC-PAD (Wu et al., 2012; Zhao et al., 2013), the chemical composition of cinobufacini capsule and injection has never been compared. Moreover, we have found that the anti-tumor effect of cinobufacini capsule and injection was quite different, which also has not been distinguished in their clinical application. It is also inferred that the *in vivo* effective substance of cinobufacini capsule and injection would be different. Unfortunately, the comparative pharmacokinetics (PK) study of cinobufacini capsule and injection has never been reported. As bufadienolides are major components in cinobufacini and toad venom preparations, previous studies have focused on the pharmacokinetic behaviors of the major bufadienolides such as resibufogenin, bufalin, gamabufotalin, arenobufagin, and bufotalin after the administration of Shexiang Baoxin pill (Huang et al., 2015). In addition, the difference in pharmacokinetics of cinobufotalin between normal and diethylnitrosamine-injured rats has also been studied, which indicated that the pharmacokinetic behaviors of cinobufotalin will be altered in rats with HCC (Zhang et al., 2019). All of these references would provide some clues for the pharmacokinetics study of cinobufacini capsule and injection.

**FIGURE 1:**
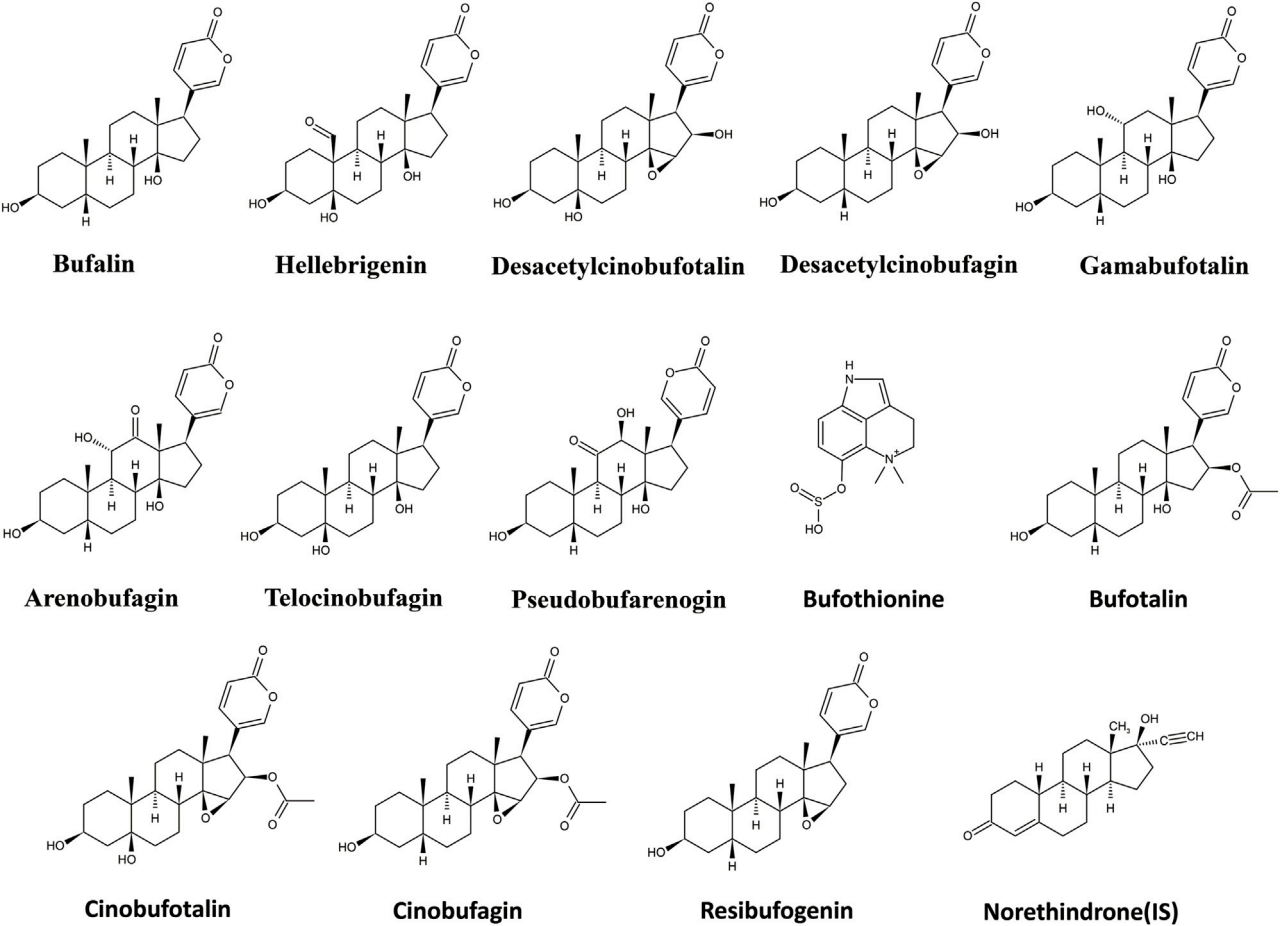
Chemical structures of 13 analytes in cinobufacini and internal standards (IS).

UPLC-MS/MS is the best tool for quantitative analysis for targeted analytes due to high selectivity and sensitivity (Abdelhameed et al., 2019; Al-Shakliah et al., 2020; Attwa et al., 2020). Furthermore, extraction methodology is also a crucial step to remove matrix constituent interferences and target compound extraction. In the present study, a rapid, selective, and efficient UPLC-MS/MS method for the simultaneous determination of bufothionine, hellebrigenin, bufalin, gamabufotalin, telocinobufagin, cinobufagin, arenobufagin, cinobufotalin, desacetylcinobufotalin, bufotalin, pseudobufarenogin, resibufogenin, and desacetylcinobufagin in the rat plasma was developed and fully validated to evaluate the comparative pharmacokinetics of cinobufacini capsule and injection. The chemical structures of the 13 analytes are shown in Figure 1. The pharmacokinetic parameters of nine compounds following the oral administration of cinobufacini capsule (0.9, 1.8, 3.6 g/kg) in rats have been calculated, while only bufothionine, telocinobufagin, bufalin, and arenobufagin could be detected in the rat plasma cinobufacini injection group. The present study for the first time revealed the significant difference in pharmacokinetic behaviors between cinobufacini capsule and injection, which would provide valuable information to discover the potential pharmacodynamic ingredients of cinobufacini capsule and injection.

## 2 Materials and Methods

### 2.1 Chemicals and Reagents

Cinobufacini injection (200504-1) was purchased from Anhui China Resources Jinchan Pharmaceutical Co., Ltd., and cinobufacini (0K01) capsules were purchased from Shaanxi Dongtai Pharmaceutical Co., Ltd. The standards (purity ≥ 98%) bufothionine (BTI), hellebrigenin (HBG), bufalin (BF), gamabufotalin (GBL), telocinobufagin (TCG), cinobufagin (CBG), arenobufagin (ABG), cinobufotalin (CBT), desacetylcinobufotalin (DCT), bufotalin (BL), pseudobufarenogin (PBG), resibufogenin (RBG), desacetylcinobufagin (DCG), and norethisterone (IS) were all purchased from Hongyong Biotechnology Co., Ltd. (Shanghai, China). Formic acid (98% purity), acetonitrile, and methanol of MS grade were purchased from Sinopharm Chemical Reagent Co., Ltd. (Shanghai, China) and Merck (Darmstadt, Germany).

### 2.2 Animal Handing

Male Sprague–Dawley rats (weighing 200–220 g) of SPF grade were offered by Sippr/BK laboratory Animal, Corp., Ltd. (Shanghai, China) and fed in the Laboratory Animal Center of the Shanghai University of Traditional Chinese Medicine. All the rats were kept under environmentally controlled conditions with constant temperature (22–24°C) and humidity (60–65%). The animal experiment program in the present research was approved by the Animal Committee of the Shanghai University of Traditional Chinese Medicine (PZSHUTCM210618012).

### 2.3 Comparative Pharmacokinetic Studies of Cinobufacini Capsule and Injection

#### 2.3.1 Instrumentation and Chromatographic Conditions

##### 2.3.1.1 Liquid Chromatography

An Agilent 1290 liquid chromatographic system includes a G4220A quaternary pump, a G1326C column incubator, a G4226A automatic sampler, and a G1330B degasser. The column temperature was set to 35°C. A measure of 5 µL of samples were separated in an Agilent SB-C18 column (2.1 mm ×50 mm, 1.8 μm) and eluted at 0.3 ml/min, followed by gradient elution with water (0.1% V/V formic acid) (A) and acetonitrile (B) (0–1 min, 20%–20% B; 1–3.5 min, 20%–80% B; 4–4.1 min, 80%-20% B; 4.1–6 min, 20% B). The total run time was 6 minutes.

##### 2.3.1.2 Mass Spectrometric Conditions

An Agilent 1290 Infinity series UPLC system was combined with an Agilent 6460 series triple quadrupole mass spectrometer (Agilent Technologies, Santa Clara, CA, United States) to quantify all 13 analytes in an ESI-positive ionization mode. Quantitative analysis was performed in an MRM mode. The mass spectrum conditions were as follows: capillary voltage, 4000V; drying gas (N_2_), gas flow rate: 10 L/min; sprayer, 30 psi; gas temperature 350°C; The delta EMV (+) 200 V; collision gas (N_2_); dwell time, 25 ms. The optimized parent ion, product ion, fragmentor, and collision energy of all analytes are shown in Table 1. Agilent’s MassHunter Workstation software was used for data collection, peak area calculation, and quantitative analysis.

**TABLE 1:** Monitoring parameters of the mass spectrometer detector.

Compound	Parent ion	Product ion	Fragmentor (V)	Collision energy (eV)	Retention time (min)
BF	387.3	351.2	135	21	3.35
BL	445.3	349.3	200	20	3.08
HBG	417.0	344.7	165	21	2.69
CBT	459.0	362.8	185	18	3.17
CBG	443.0	365.0	185	15	3.57
DCT	417.0	363.1	180	20	2.69
GBL	403.0	252.9	210	24	2.33
ABG	417.0	398.8	205	29	2.66
TCG	403.0	348.9	210	21	2.99
PBG	417.0	398.9	230	31	2.24
RBG	385.0	366.8	150	15	3.60
DCG	401.0	105.2	145	45	3.04
BTI	283.1	202.8	110	16	0.66
IS	299.0	109.1	110	34	3.49

#### 2.3.2 Preparation of the Standard Solutions and Quality Control Samples

Standard substances of 13 compounds were accurately weighed in appropriate amounts and dissolved with methanol in a 10-ml volumetric flask to prepare the stock solutions. Then, 1 ml of each stock solution of 13 compounds was mixed to prepare the standard working solution and then diluted with methanol at eight concentration levels. Norethisterone was chosen as the internal standard (IS) to prepare its stock solution at 1 mg/ml. Then, it was diluted into an internal standard solution with a concentration of 345 ng/ml. All solutions were stored at 4°C before use. The calibration standard solution was prepared by spiking appropriate amounts of the standard working solutions into blank rat plasma, and the range of their final concentrations is shown in Table 2. The quality control (QC) samples at three different levels were also prepared following the same procedures, according to that of the calibration standard solution.

**TABLE 2:** Regression equations, linear range, and lower limit of quantification (LLOQ) of analytes in the rat plasma.

Analyte	Regression equation	Correlation coefficient (r)	Linear range (ng/ml)	LLOQ (ng/mL)
BF	Y = 0.0124X + 0.0734	0.9994	5.00–499.98	4.13
BL	Y = 0.0370X + 0.2442	0.9972	0.50–500.46	0.20
HBG	Y = 0.0003X − 0.0015	0.9987	5.00–499.70	2.54
CBT	Y = 0.0038X + 0.0049	0.9989	2.50–500.50	1.02
CBG	Y = 0.0117X + 0.0191	0.9969	0.50–500.20	0.28
DCT	Y = 0.0051X + 0.0296	0.9989	0.50–499.90	0.38
DCG	Y = 0.0098X + 0.2881	0.9992	5.00–500.55	4.17
GBL	Y = 0.0026X + 0.0222	0.9967	0.50–499.84	0.44
ABG	Y = 0.0111X + 0.0372	0.9979	5.00–499.69	2.81
TCG	Y = 0.0062X + 0.0222	0.9975	1.00–500.50	0.72
PBG	Y = 0.0060X + 0.0082	0.9981	5.00–500.40	3.12
RBG	Y = 0.0040X + 0.0260	0.9996	2.50–499.16	1.02
BTI	Y = 0.0015X + 0.0051	0.9983	5.00–499.80	4.84

#### 2.3.3 Biosample Preparation

A measure of 50 µL of the plasma sample was mixed with 10 µL of the internal standard solution (345 ng/ml) in a 1.5-ml centrifuge tube and vortexed for 5 min. The samples were extracted by 500 µL of ethyl acetate for 5 min and centrifuged at 4°C for 10 min (18000 rpm). All the supernatants were collected into another 1.5-ml centrifuge tube, dried under a gentle stream of nitrogen at 37°C, and then added with 50 µL of 80% methanol for resolution. The resolution samples were centrifuged at 4°C for 10 min (18000 rpm), and 5 µL supernatant was extracted and injected for analysis.

#### 2.3.4 Method Validation

Validation parameters of the method included selectivity, linearity, precision and accuracy, recovery, matrix effect, and stability. All tests were performed in accordance with the U. S. Food and Drug Administration guidelines for the validation of bioanalytical methods (The Food And Drug Administration., 2018).

##### 2.3.4.1 Selectivity

The specificity of the present method was evaluated by chromatograms of six different batches of blank rat plasma samples, blank samples spiked with all 13 analytes and IS, and rat plasma samples after drug administration of 0.5 h to check for endogenous interference.

##### 2.3.4.2 Linearity and Lower Limit of Quantification

The calibration curve was generated by measuring standard plasma samples at eight concentration levels, and the linear relationship between the peak area ratio of the 13 analytes to the internal standard and the analyte concentrations in the calibrated plasma samples was constructed. The lower limit of quantification (LLOQ) is defined as the lowest quantifiable calibration concentration with a signal-to-noise ratio (S/N) of 10, with an acceptable accuracy within ±20%.

##### 2.3.4.3 Precision and Accuracy

The intra- and inter-day precision and accuracy were evaluated by determining QC samples at three different concentrations (six replicates for each concentration level) in one day and over three consecutive days. The concentrations were determined using calibration curves obtained daily. The intra-day and inter-day precisions were evaluated as a variability with a relative standard deviation percentage (RSD) of <15%, and accuracy was expressed as a relative error percentage (RE, %) within ±15%.

##### 2.3.4.4 Recovery Efficiency and Matrix Effect

The extraction recoveries were determined at three QC levels by comparing the peak area of the plasma samples spiked with all 13 analytes added before extraction to that of plasma samples spiked with analytes after extraction. Matrix effects were measured at three QC levels by comparing the peak area of the extracted blank plasma samples (from six different batches of rat plasma) with that of the corresponding pure standard solution. The extraction recoveries and matrix effects were acceptable when their RSD <15%.

##### 2.3.4.5 Stability

The stability of QC samples in six replicates at three concentrations (low, medium, and high levels) was analyzed under different storage conditions: the post-treatment stability was analyzed after storage in an autosampler (4°C) for 24 h. Freeze–thaw stability was investigated after three freeze–thaw cycles (−80°C to room temperature as one cycle). Long-term stability was assessed by analyzing QC samples kept at −80°C for 30 days. The concentrations of the analytes in the plasma samples and the QC samples were calculated by the calibration standard curve prepared each day. The stabilities of analytes were acceptable when the accuracy (RE, %) and precision (RSD, %) of all QC samples in all storage conditions were within ±15% compared to nominal concentrations.

#### 2.3.5 Application to Pharmacokinetic Analysis

Twenty-four male Sprague–Dawley rats (weigh 200–220 g) were divided into four groups with six rats in each group randomly, including three capsule groups at three different doses and one injection group. In the instructions for cinobufacini capsule and injection, the clinical dosage for adults is 2 g per 70 kg orally and 20 ml per 70 kg intravenously, converting to rat doses of 180 mg/kg and 1.8 ml/kg, respectively.

In the pre-experiments, we compared cinobufacini capsule of 180 mg/kg (one-time clinical dose) and 900 mg/kg (five times of clinical dose) and Cinobufacini injection of 1.8 ml/kg, but concentrations of some compounds in plasma were too low and difficult to be quantified. Therefore, 0.9, 1.8, 3.6 g/kg (5, 10, and 20 times of clinical dose), and 9 ml/kg (five times of clinical dose) were chosen as administered doses of cinobufacini capsule and injection, which could detect more active components in the rat plasma and could demonstrate the pharmacokinetic characteristics of all compounds sufficiently. The experimental rats were led to fast the night and were free to get access to water for 12 h before the pharmacokinetic study. After administration, blood samples (approximately 150 μL) of rats were collected via retro-orbital sinus into heparinized centrifuge tubes at 0.05, 0.083, 0.25, 0.5, 0.75, 1, 2, 4, 6, 8, and 10 h. After centrifugation at 5,000 rpm for 10 min, the plasma was transferred to 1.5-ml polypropylene tubes and stored at −80°C until UPLC-MS/MS analysis.

### 2.4 Data Processing and Statistical Analysis

GraphPad Prism software (version 8.0.2) was used to draw the mean plasma concentration–time curves of all analytes. The pharmacokinetic parameters were calculated by DAS 3.2.8 software. Meanwhile, the pharmacokinetic data on rats were presented as mean ± standard deviation (SD). Differences of pharmacokinetic parameters between groups were analyzed by the *t*-test using SPSS software (version 26.0). Differences were considered significant statistically when the *p*-values were <0.05 and very significant statistically when the *p*-values were <0.01.

## 3 Results

### 3.1 Optimization of UPLC-MS/MS Conditions

The standard solution of each compound (500 ng/ml) was analyzed by the mass spectrometer with spectra from m/z 50 to 1000 in order to optimize the mass spectrometry conditions. In the positive ion mode, all 13 analytes could produce a strong mass spectral response. The mass spectrum parameters such as capillary voltage and collision energy were optimized in the multiple reaction monitoring (MRM) model, and the parent ions, product ions, fragmentors (V), and collision energies (CE) of all analytes are revealed in Table 1. The mobile phase for gradient elution was optimized, while acetonitrile could obtain good separation efficiency for the majority of all analytes in 6 min by using an Agilent SB C18 column (2.1 mm× 50 mm, 1.8 μm). Moreover, we evaluated the effects of different proportions of formic acid in mobile phases to maximize the mass spectrum response of the compounds, indicating that acetonitrile–0.1% formic acid solution could be used as a gradient elution condition to obtain a better peak shape.

### 3.2 Optimization of Extraction Conditions

The protein precipitation (PPT) method using methanol, acetonitrile, or their mixture and liquid–liquid extraction (LLE) with ethyl acetate has been used to extract 13 compounds and IS from rat plasma simultaneously. The protein precipitation (PPT) method by using methanol, acetonitrile, and their mixture was not suitable, while the extraction recoveries of all analytes were low. Also, the LLE method with ethyl acetate could provide higher extraction recoveries than 50% for all analytes and also offer better selectivity. Hence, LLE with ethyl acetate was used for sample pretreatment in this study.

#### 3.2.1 Method Validation

##### 3.2.1.1 Selectivity

**FIGURE 2:**
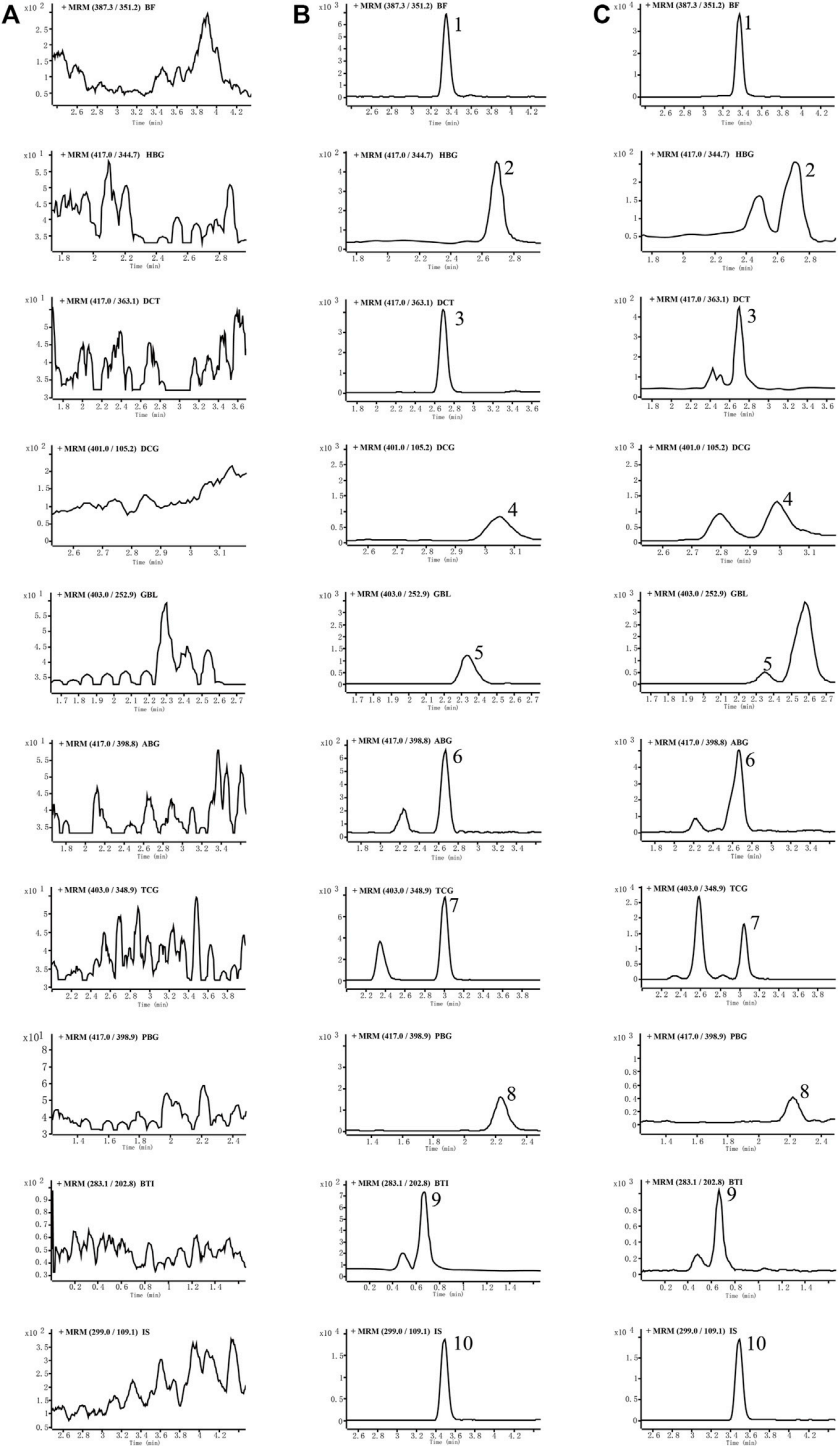
Representative MRM chromatograms of nine analytes and internal standards (IS), in rat plasma: blank rat plasma **(A)**, blank rat plasma spiked with the analytes and IS **(B)**, and rat plasma samples at 0.5 h after drug administration **(C)**. Peak 1: bufalin, 2: hellebrigenin, 3: desacetylcinobufotalin, 4: desacetylcinobufagin, 5: gamabufotalin, 6: arenobufagin, 7: telocinobufagin, 8: pseudobufarenogin, 9: bufothionine, and 10: norethisterone (IS).

No endogenous interference was found in the retention time of analytes and internal standard in blank plasma of six different rats, which proved the specificity of this method. Typical chromatograms of blank plasma, blank plasma with nine analytes added, and plasma samples of normal rats 0.5 h after oral are shown in Figure 2 to describe that there were no endogenous interferences for their quantification. The MRM mass spectra with the corresponding fragmentation for all analytes are shown in Supplementary Figure S1 of the supplementary material. Another four analytes including BL, CBT, CBG, and RBG could not be detected in the rat plasma in both cinobufacini capsules and injection groups, and typical chromatograms of blank plasma and blank plasma with four analytes added were also compared and are shown in Supplementary Figure S2 of the supplementary material. Blank plasma added with standard samples was freshly prepared according to the QC samples in a low level, with a concentration of 5.00 ng/ml for BF; 5.00 ng/ml for BL; 5.00 ng/ml for HBG; 5.00 ng/ml for CBT; 5.00 ng/ml for CBG; 5.00 ng/ml for DCT; 5.00 ng/ml for DCG; 5.00 ng/ml for GBL; 5.00 ng/ml for ABG; 5.00 ng/ml for TCG; 5.00 ng/ml for PBG; 4.99 ng/ml for RBG, and 5.00 ng/ml for BTI.

##### 3.2.1.2 Linear and Quantitative Lower Bound

By weighted (1/x^2^) least-squares linear regression, the relationship curve between the peak area ratio (y) of each analyte to IS and the corresponding nominal concentration (x) of the analyte was plotted. The correlation coefficient for all calibration curves above 0.9967 showed good linearity for quantitation of all analytes in rat plasma. The established method was verified to meet the requirements of the quantitative determination for pharmacokinetic studies, and results of the calibration curves, linear ranges, and correlation coefficients are shown in Table 2.

##### 3.2.1.3 Precision and Accuracy

Intra-day and daytime accuracy (relative standard deviation, RSD) is less than 14.3%, and accuracy (relative error, RE) is less than 13.0%, as summarized in Table 3. All results were within acceptable standards, according to the guidelines for bioassay methods.

**TABLE 3:** Summary of accuracy, precision, recovery, and matrix effect of the analytes in rat plasma (*n* = 6).

Analyte	Concentration (ng/ml)	Precision and accuracy				Recovery (%)		Matrix effect (%)	
		Intra-day (%)		Inter-day (%)					
		RSD	RE	RSD	RE	Mean	RSD	Mean	RSD
BF	5.00	9.8	6.4	3.3	6.1	63.1	6.9	94.8	3.7
	50.00	6.5	−0.4	3.4	−1.4	58.4	4.3	88.7	1.8
	249.99	7.6	0.2	4.1	−1.6	63.9	9.8	93.1	11.2
BL	5.00	9.5	−2.0	4.2	1.2	52.8	9.1	105.2	10.9
	50.05	7.1	2.1	5.8	−3.5	71.9	3.2	109.7	1.1
	250.23	5.1	7.2	2.2	6.8	90.9	9.2	110.6	10.0
HBG	5.00	7.4	10.2	5.0	4.5	54.2	4.2	109.9	10.7
	49.97	11.0	−2.8	4.1	0.2	69.2	4.6	113.8	4.6
	249.85	10.0	−2.4	4.1	0.8	83.7	7.7	110.9	7.7
CBT	5.00	11.3	2.4	3.7	6.9	58.2	13.9	89.1	11.6
	50.05	7.0	−0.8	6.7	7.2	55.6	6.8	92.0	5.6
	250.25	8.8	4.2	3.0	4.5	65.4	4.4	96.5	11.8
CBG	5.00	9.3	5.0	4.4	0.2	57.3	9.7	110.9	3.5
	50.02	7.8	6.7	8.1	−2.0	55.6	3.3	113.4	5.7
	250.10	9.4	0.2	2.7	2.2	71.3	8.2	97.3	3.9
DCT	5.00	9.4	5.7	2.8	5.0	97.8	6.6	89.4	4.2
	49.99	8.5	2.2	7.1	−4.1	86.7	3.2	96.9	3.8
	249.95	12.3	−12.5	9.6	−5.2	92.3	2.4	107.0	1.8
DCG	5.00	7.9	11.4	7.9	2.4	57.8	4.7	111.4	13.0
	50.06	14.3	−2.6	3.5	−3.5	82.6	3.4	106.7	3.0
	250.28	9.0	−5.5	6.1	1.5	96.6	9.5	108.9	10.3
GBL	5.00	10.4	11.3	0.6	10.5	52.7	4.8	106.4	6.5
	49.98	7.6	−7.1	3.0	−3.8	56.5	4.0	107.8	2.7
	249.92	8.9	−8.6	4.7	−3.3	72.9	0.7	105.1	4.6
ABG	5.00	6.6	12.6	6.9	6.8	61.9	4.0	111.2	2.0
	49.97	10.2	−3.6	2.0	−5.8	62.2	5.8	110.2	5.8
	249.84	7.1	−5.3	2.5	−2.6	80.3	3.4	109.0	3.7
TCG	5.00	9.8	11.9	1.5	11.6	52.0	6.5	104.4	3.9
	50.05	7.7	−2.0	4.3	−2.9	69.6	1.8	100.8	0.9
	250.25	3.9	−4.3	3.9	−2.4	87.2	9.7	103.0	10.4
PBG	5.00	5.8	13.0	5.1	8.1	64.7	11.7	95.8	13.7
	50.04	6.7	−0.3	3.9	1.7	65.4	4.2	104.6	7.4
	250.20	10.0	4.7	6.6	2.6	80.7	1.0	92.3	5.5
RBG	4.99	7.9	4.1	4.4	2.1	80.2	3.7	107.4	0.6
	49.92	9.4	6.1	5.7	1.1	68.8	2.7	110.6	0.9
	249.58	8.2	−1.8	0.8	−1.1	89.0	6.0	96.5	4.6
BTI	5.00	9.7	3.4	2.8	3.4	4.3	5.1	93.7	10.7
	49.98	9.0	−6.1	3.8	−4.0	4.0	12.1	90.2	8.1
	249.90	11.7	−3.2	3.8	1.2	6.2	4.7	90.8	10.3

##### 3.2.1.4 Recovery and Matrix Effect

The average extraction recoveries and matrix effects of 13 analytes at three quality control levels are summarized in Table 3, while the recoveries of 12 analytes except BTI at three concentration levels in rat plasma samples ranged from 52.0 % to 97.8%, and the matrix effect ranged from 88.7% to 113.8%. But the extraction recovery of BTI ranged from 4.0% to 6.2%, which was out of requirement of method validation. Most sample pretreatment methods have been used to increase its extraction recovery rate, but it does not work. The pharmacokinetics of BTI has never been reported in previous studies. Fortunately, the concentration of BTI in rat plasma is high enough for quantitation. We hope it would be solved in further research.

##### 3.2.1.5 Stability

The stability of 13 analytes at three quality control levels is summarized in Table 4. The accuracy (RE, %) of all QC samples in the stability test ranged from −12.6% to 13.3% with precision (RSD, %) in the range of 2.1%–13.5%. It indicated that analytes in rat plasma remained stable in plasma samples under the following conditions: post-treatment storage in an autosampler (4°C) for 24 h, three freeze–thaw cycles, and at −80°C for 30 days.

**TABLE 4:** Stability of 13 analytes in the rat plasma at three QC levels (*n* = 6).

Analyte	Concentration (ng/ml)	Stability during 24 h at 4°C		Three freeze–thaw cycles		−80°C for 30 days	
		RSD (%)	RE	RSD (%)	RE (%)	RSD (%)	RE (%)
BF	5.00	12.0	3.3	7.9	−7.7	5.7	8.7
	50.00	5.3	−8.6	7.0	−10.1	4.3	−12.6
	249.99	12.5	−8.5	12.0	0.8	7.5	1.4
BL	5.00	5.4	11.4	9.0	3.4	9.0	2.3
	50.05	6.3	5.0	7.7	3.1	7.7	2.1
	250.23	6.2	5.7	3.4	7.1	3.4	6.0
HBG	5.00	7.2	10.2	8.9	9.3	7.4	−5.0
	49.97	8.1	−4.7	12.0	−2.1	8.0	8.9
	249.85	12.5	0.8	8.4	1.5	13.4	−1.3
CBT	5.00	7.6	9.8	10.0	4.3	9.4	−0.3
	50.05	7.9	7.7	5.1	13.2	9.4	4.1
	250.25	12.9	4.8	10.5	12.0	7.6	−8.7
CBG	5.00	8.1	4.8	6.4	4.8	6.4	3.7
	50.02	7.8	−3.2	7.6	0.0	7.6	−1.0
	250.10	11.3	0.6	12.2	3.4	12.2	2.3
DCT	5.00	5.5	11.4	11.2	3.7	13.2	2.4
	49.99	11.3	7.3	6.5	12.1	5.6	12.7
	249.95	7.0	9.6	7.9	7.3	8.6	8.7
DCG	5.00	13.5	1.5	11.3	3.0	11.3	1.9
	50.06	6.4	6.7	8.0	4.9	8.0	3.8
	250.28	4.5	4.6	5.9	−2.8	5.9	−3.8
GBL	5.00	6.2	12.0	2.1	13.2	2.2	8.3
	49.98	10.6	10.9	5.4	10.7	6.1	12.4
	249.92	7.9	5.4	7.7	−0.6	8.0	−4.9
ABG	5.00	12.8	7.4	10.3	2.5	10.2	7.1
	49.97	12.5	−5.3	12.5	3.3	10.7	8.3
	249.84	11.2	−4.1	11.1	0.7	11.9	2.3
TCG	5.00	11.2	−0.3	10.6	5.8	8.7	0.9
	50.05	12.8	−4.2	5.7	0.0	7.6	3.8
	250.25	3.7	−4.7	8.2	-6.6	7.9	−2.1
PBG	5.00	11.0	6.6	11.1	4.4	6.9	8.3
	50.04	9.9	12.0	5.0	13.3	6.4	7.5
	250.20	9.7	10.2	11.5	7.7	11.6	4.7
RBG	4.99	7.1	−6.4	9.1	−0.3	9.1	−1.3
	49.92	8.9	3.5	11.7	3.3	11.7	2.3
	249.58	9.5	−3.3	7.5	−1.9	7.5	−2.8
BTI	5.00	8.1	−0.3	10.4	−3.4	9.7	1.7
	49.98	7.7	−9.3	5.3	2.5	9.0	−4.1
	249.90	5.0	−5.6	5.3	5.0	9.0	3.0

## 4 Discussion

**FIGURE 3:**
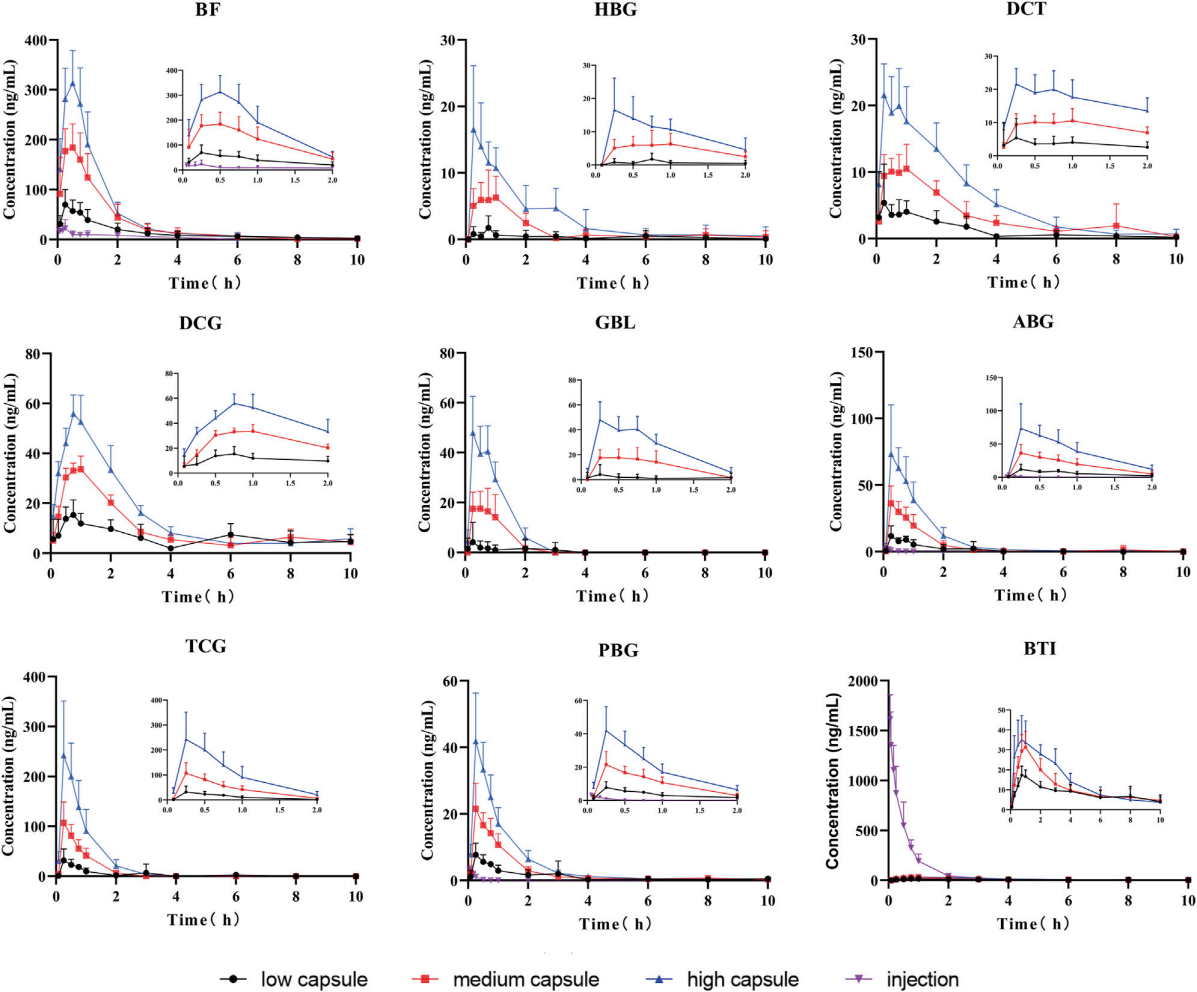
Mean concentration–time curves of nine compounds in rat plasma after the oral administration of cinobufacini capsule (0.9, 1.8, and 3.6 g/kg) and intravenous administration of cinobufacini injection at a dose of 9 ml/kg to male SD rats (mean ± SD, *n* = 6).

The established UPLC-MS/MS method has been successfully applied for the pharmacokinetic study of cinobufacini capsule and injection in rats. The concentrations of BF, HBG, DCT, DCG, GBL, ABG, TCG, BTI, and PBG in rat plasma after the oral administration of cinobufacini capsule could be determined. The concentrations of BTI, BF, ABG, and PBG in rat plasma after the intravenous administration of cinobufacini injection through the tail vein could be determined. The mean plasma concentration–time curves are presented in Figure 3. The pharmacokinetic parameters including the maximum plasma concentration (C max), area under the concentration–time curve (AUC) and mean residence time (MRT), the half-life life (t _1/2z_), and clearance (CL/F) are summarized in Tables 5, 6. The pharmacokinetic parameters in the cinobufacini capsule group were calculated by a two-compartmental model by DAS 3.2 data analysis software.

**TABLE 5:** Pharmacokinetic parameters of the target analytes after the oral administration of cinobufacini capsule (0.9, 1.8, and 3.6 g/kg) and intravenous administration of cinobufacini injection at a dose of 9 ml/kg to male SD rats (mean ± SD, *n* = 6).

Analyte	Groups	C_max_ (μg/L)	T_max_(h)	T_1/2z_(h)	Vz/F (L/kg)	CLz/F (L/h/kg)	AUC_0-t_ (μg/L*h)	AUC_0-∞_(μg/L*h)	MRT_0-t_(h)	MRT_0-∞_(h)
BF	Low	74.70 ± 30.70	0.25 ± 0.00	1.09 ± 0.91	1.81 ± 0.56	1.57 ± 0.69	139.28 ± 71.90	143.06 ± 79.36	2.04 ± 0.62	2.19 ± 0.90
	Medium	189.04 ± 43.18^A^	0.43 ± 0.12^A^	1.20 ± 0.62	2.15 ± 1.21	1.29 ± 0.43	310.28 ± 131.39^a^	316.94 ± 133.54^a^	1.45 ± 0.24^a^	1.59 ± 0.49
	High	322.02 ± 55.54^AB^	0.46 ± 0.17^a^	1.27 ± 0.72	3.02 ± 2.01	1.64 ± 0.36	456.75 ± 91.19^Ab^	460.64 ± 90.36^Ab^	1.39 ± 0.43^a^	1.49 ± 0.55
HBG	Low	2.47 ± 1.38	0.94 ± 0.75	1.02 ± 0.60	14.16 ± 3.88	14.2 ± 7.01	3.97 ± 1.73	4.21 ± 2.23	3.59 ± 1.63	3.81 ± 1.99
	Medium	8.36 ± 2.92^A^	0.68 ± 0.35	1.34 ± 1.09	11.11 ± 5.04	6.63 ± 3.60	13.73 ± 4.30^A^	13.84 ± 5.40^A^	2.28 ± 1.26	2.55 ± 1.53
	High	18.16 ± 5.84^AB^	0.39 ± 0.20	0.63 ± 0.10	6.35 ± 2.55^A^	7.06 ± 2.71^a^	31.07 ± 11.41^AB^	31.07 ± 11.41^AB^	2.14 ± 0.83	2.14 ± 0.82
DCT	Low	6.936 ± 5.35	0.87 ± 0.63	0.83 ± 0.25	5.16 ± 2.57	3.96 ± 1.01	12.98 ± 3.69	13.43 ± 3.76	2.53 ± 0.99	2.95 ± 1.78
	Medium	12.26 ± 3.11^A^	0.61 ± 0.32	1.97 ± 1.36	8.27 ± 5.18	3.02 ± 0.78	34.24 ± 10.43^A^	35.70 ± 12.05^A^	2.68 ± 0.88	3.10 ± 0.92
	High	22.19 ± 5.19^AB^	0.39 ± 0.20	1.53 ± 0.82	5.59 ± 3.29	2.55 ± 0.72^a^	61.18 ± 16.14^AB^	62.69 ± 16.75^AB^	2.28 ± 0.37	2.53 ± 0.58
DCG	Low	17.20 ± 4.77	0.83 ± 0.20	1.22 ± 0.81	2.8 ± 1.71	0.78 ± 0.15	61.73 ± 7.80	66.97 ± 15.27	3.95 ± 0.73	4.74 ± 1.56
	Medium	35.61 ± 2.59^A^	0.89 ± 0.13	4.76 ± 0.52^A^	5.62 ± 0.68^a^	0.72 ± 0.35	100.27 ± 12.45^A^	112.49 ± 14.95^A^	3.00 ± 0.63^a^	4.86 ± 0.46
	High	56.62 ± 7.79^AB^	0.82 ± 0.12	2.09 ± 0.60^B^	2.79 ± 1.51^b^	1.03 ± 0.22	147.67 ± 30.20^AB^	150.43 ± 29.98^AB^	2.44 ± 0.41^A^	2.65 ± 0.44^AB^
GBL	Low	13.28 ± 5.28	0.25 ± 0.00	0.37 ± 0.00	4.75 ± 0.98	9.00 ± 1.86	10.80 ± 2.45	13.59 ± 3.08	0.41 ± 0.11	0.64 ± 0.16
	Medium	21.48 ± 7.57^A^	0.46 ± 0.23	0.28 ± 0.26	3.03 ± 2.02	12.57 ± 5.12	21.85 ± 11.62^A^	23.38 ± 15.04^A^	0.73 ± 0.10	0.79 ± 0.22
	High	49.50 ± 10.94^AB^	0.36 ± 0.20	0.30 ± 0.16	3.74 ± 1.66	9.18 ± 2.66	54.00 ± 11.97^AB^	54.52 ± 12.38^AB^	0.83 ± 0.12	0.86 ± 0.14
ABG	Low	13.80 ± 6.40	0.45 ± 0.27	0.82 ± 0.66	8.01 ± 5.68	6.53 ± 2.41	15.50 ± 4.73	15.54 ± 4.73	1.44 ± 0.58	1.47 ± 0.60
	Medium	36.67 ± 13.05^A^	0.32 ± 0.12	0.64 ± 0.16	4.20 ± 1.25	4.27 ± 1.06	44.08 ± 10.59^A^	45.88 ± 13.11^A^	1.49 ± 1.03	1.74 ± 1.29
	High	77.92 ± 24.22^AB^	0.32 ± 0.12	0.89 ± 0.64	5.66 ± 3.45	4.65 ± 1.65	87.27 ± 27.35^AB^	87.61 ± 27.83^AB^	1.20 ± 0.26	1.23 ± 0.31
TCG	Low	35.25 ± 21.04	0.36 ± 0.20	0.16 ± 0.01	1.75 ± 0.60	7.71 ± 2.54	24.49 ± 10.27	24.49 ± 10.27	0.64 ± 0.06	0.64 ± 0.06
	Medium	109.08 ± 41.32^A^	0.29 ± 0.09	0.21 ± 0.11	1.13 ± 0.37	3.97 ± 1.13^A^	89.70 ± 23.80^A^	89.93 ± 24.11^A^	0.73 ± 0.11	0.74 ± 0.11
	High	253.40 ± 83.80^AB^	0.32 ± 0.12	0.53 ± 0.02^AB^	2.76 ± 1.67	3.62 ± 1.71^A^	217.14 ± 82.40^AB^	217.14 ± 82.40^AB^	0.83 ± 0.13^A^	0.83 ± 0.13^A^
PBG	Low	9.30 ± 3.042	0.25 ± 0.00	1.52 ± 0.98	7.15 ± 2.16	2.01 ± 0.59	13.29 ± 4.42	13.80 ± 5.40	2.40 ± 0.63	2.76 ± 1.00
	Medium	24.50 ± 7.32^A^	0.40 ± 0.22	1.03 ± 0.61	2.65 ± 1.51^a^	1.85 ± 0.48	28.43 ± 6.26^A^	28.55 ± 6.25^A^	1.49 ± 0.08^a^	1.54 ± 0.11^a^
	High	42.84 ± 10.23^AB^	0.32 ± 0.12	1.96 ± 0.67	5.65 ± 1.61^b^	2.20 ± 0.48	47.26 ± 9.40^AB^	47.70 ± 9.78^AB^	1.47 ± 0.39^a^	1.57 ± 0.39^a^

^a^ *p* < 0.05, ^A^ *p* < 0.01, vs. low-dose group; ^b^ *p* < 0.05., ^B^ *p* < 0.01, vs. medium-dose group.

**TABLE 6:** Pharmacokinetic parameters of bufothionine after the oral administration of cinobufacini capsule (0.9, 1.8, and 3.6 g/kg) and intravenous administration of cinobufacini injection at a dose of 9 ml/kg to male SD rats (mean ± SD, *n* = 6).

Analyte	Groups	C_max_ (μg/L)	T_max_ (h)	T_1/2z_ (h)	Vz/F (L/kg)	CLz/F (L/h/kg)	AUC_0-t_ (μg/L*h)	AUC_0-∞_ (μg/L*h)	MRT_0-t_(h)	MRT_0-∞_(h)
BTI	low	19.20 ± 3.26	0.88 ± 0.14	5.18 ± 4.01	5.69 ± 3.66	0.95 ± 0.33	84.72 ± 15.32	121.38 ± 41.28	4.02 ± 0.61	6.85 ± 3.58
	medium	33.29 ± 8.328^A^	0.86 ± 0.20	4.79 ± 3.86	8.89 ± 5.23	1.46 ± 0.30	114.90 ± 15.20^A^	151.86 ± 47.45	3.45 ± 0.55	5.34 ± 2.28
	high	37.00 ± 11.634^A^	0.68 ± 0.12	2.26 ± 1.09	9.03 ± 4.54	2.88 ± 0.74	144.87 ± 39.14^A^	154.80 ± 43.50	3.09 ± 0.32	3.76 ± 0.68
	Injection	1628.02 ± 261.07	−	0.60 ± 0.07	1.08 ± 0.22	1.25 ± 0.23	860.78 ± 152.62	861.28 ± 152.67	0.62 ± 0.04	0.62 ± 0.04

^a^ *p* < 0.05, ^A^ *p* < 0.01, vs. low-dose group; ^b^ *p* < 0.05, ^B^ *p* < 0.01, vs. medium-dose group.

After gavage administration, HBG, DCT, GBL, ABG, TCG, and PBG reached maximum plasma concentrations (C_max_) within 30 min, and BF, BTI, and DCG reached maximum plasma concentrations within 60 min. It suggested that these nine analytes were immediately absorbed and distributed into blood. Moreover, T_1/2_, MRT_0-∞,_ and MRT_0-t_ of the nine analytes ranged from 0.16 to 4.86 h, indicating that the elimination rates of all analytes were metabolized and eliminated fast *in vivo*. The T_1/2z (h)_, MRT_0-t_ (h), and CLz/F (μg/L*h) values of the compounds were between 0.16 ± 0.01 and 4.76 ± 0.52, between 0.41 ± 0.11 and 3.95 ± 0.73, and between 0.95 ± 0.33 and 14.16 ± 3.88, respectively. The CLz/F values of GBL and HBG were higher than other compounds, indicating that the elimination rates of most of the analytes in rat plasma were rapid, and the elimination rates of GBL and HBG were the fastest in nine compounds. C_max_, AUC_0-t,_ and AUC_0-∞_ of nine compounds were dose-dependently increased and showed significant difference among three dosage groups (*p* < 0.05; *p* < 0.01). BF had the highest C_max_ value and the highest AUC_0-t_ value compared with the other compounds, indicating that BF had a high level exposure in rat plasma. C_max_, AUC_0-t_, and AUC_0-∞_ values of TBG, BF, DCG, and ABG were relatively higher than other compounds, suggesting that these four compounds could be the major effective components in cinobufacini capsule.

Furthermore, the comparative pharmacokinetic behaviors of cinobufacini capsule and injection have been compared at the same dose, which indicated that TBG, BF, DCG, and ABG were the principal active compounds in cinobufacini capsule, and only BTI was the major active compound in cinobufacini injection. Meanwhile, BF, ABG, and PBG could be detected in rat plasma after the intravenous administration of cinobufacini injection. But their concentrations in most rat plasma samples were far below the LLOQs so that their pharmacokinetic parameters could not be calculated. The mean plasma concentration–time curve of BTI could be drawn, and its pharmacokinetic parameters could be calculated (Figure 3). The C_max_ (1628.02 ± 261.07 ng/ml) and AUC_0-t_ (860.78 ± 152.62 μg/L*h) of BTI in the cinobufacini injection group were higher than those in the cinobufacini capsule group. It could be speculated that BTI is the main component in cinobufacini injection, while bufadienolides including BTI, BF, HBG, DCT, DCG, GBL, ABG, TCG, and PBG were the major components in cinobufacini capsule. Despite a few reports on the main components in Cinobufacini capsules or injection, some previous studies about the pharmacokinetic characteristics of bufadienolides in rats after the administration of a single bufadienolide or the single medicinal material *Venenum Bufonis*, which is the dry secretion of *Bufo bufo gargarizans* Cantor, have been reported. Moreover, five bufadienolides, including RBG, BF, ABG, GBL, and BL, after administration of Shexiang Baoxin pill, were also determined in rat plasma, simultaneously (Huang et al., 2015). Interestingly, the pharmacokinetic parameters of BF and ABG including T_max_, t_1/2_, and MRT were similar in both Huang’s and our studies. Meanwhile, even the pharmacokinetic parameters of BL have been calculated in Huang’s studies, but the concentration of BL is lower than 4.4 ng/ml. In our present study, the concentration of BL in plasma is too low to calculate the pharmacokinetics parameters accurately. CBG, another indicative component in *Venenum Bufonis*, was not detected in both Huang’s and our studies. However, CBG was detected in Wang’s studies with C_max_ at 4.49 ng/ml when the administration dose was 1.11 mg/kg (Wang et al., 2014). In our present study, the administration dose was calculated at 0.36 mg/kg for CBG, which was lower than the dose in Wang’s studies. So CBG could not be detected in the present study. Moreover, it has been reported that CBG could be metabolized into forms of desacetylcinobufagin and 3-epidesacetylcinobufagin, or in other forms through hydroxylation (Toma et al., 1987; He et al., 2012), which can also cause the decrease in CBG concentration in rat plasma. So the metabolism process of bufadienolides in cinobufacini preparations should be studied in further research.

To the best of our knowledge, bufadienolides and indolealkylamines are the two major ingredients of active compounds in cinobufacini, as BF, HBG, DCT, DCG, GBL, ABG, TCG, and PBG belong to bufadienolides, and BTI belongs to indolealkylamines. The different preparation processes between cinobufacini capsule and injection cause a huge difference in their components, especially the preparation process of the boiling water extraction into water-soluble injection, leading to most of the bufadienolides being removed. So the present comparative pharmacokinetic studies inferred that bufadienolides including BF, HBG, DCT, DCG, GBL, ABG, TCG, and PBG were the main bioactive components in cinobufacini capsule, while BTI is the main bioactive component in cinobufacini injection. Moreover, the antitumor effect of BTI was much less than most of bufadienolides, such as CBG and RBG (Xie et al., 2012; Qi et al., 2018). So their antitumor effects have been proven to be widely different from our other studies. So the present research would provide a more valuable information and scientific basis for further discovery of potential pharmacodynamic ingredients, clinical application, and quality control of cinobufacini capsule and injection.

## 5 Conclusion

A rapid, selective, and sensitive UPLC-MS/MS method was established and validated for the simultaneous determination of BTI, HBG, BF, GBL, TCG, CBG, ABG, CBT, DCT, BL, RBG, PBG, and DCG in rat plasma. It was fully validated *via* the linearity, precision, extraction recovery, matrix effect, and stability test. The method was successfully applied for the pharmacokinetic study of cinobufacini capsule and injection in rats, which indicated that the pharmacokinetic characteristics of cinobufacini capsules and injection were significantly different for the first time. It indicated that bufadienolides and BTI were the main bioactive components in cinobufacini capsule and injection, respectively, which would give comprehensive information for understanding the difference of pharmacodynamics between cinobufacini capsules and injection. This could facilitate further research on the action mechanism of cinobufacini capsules and injection.

## Data Availability

The original contributions presented in the study are included in the article/Supplementary Material; further inquiries can be directed to the corresponding authors.
