# Supplementary material for: Comparative Pharmacokinetics of Cinobufacini Capsule and Injection by UPLC-MS/MS
Source: Front Pharmacol. 2022 Jul 18;13:944041. doi: 10.3389/fphar.2022.944041 (PMC9343874; doi:10.3389/fphar.2022.944041)
Supplement: Supplementary file 1 [file DataSheet1.docx]

Supplementary Material


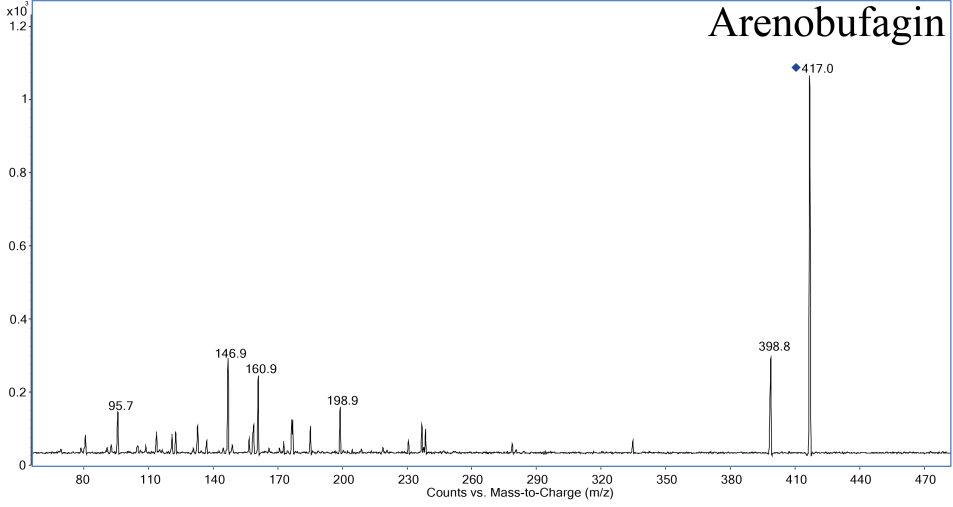


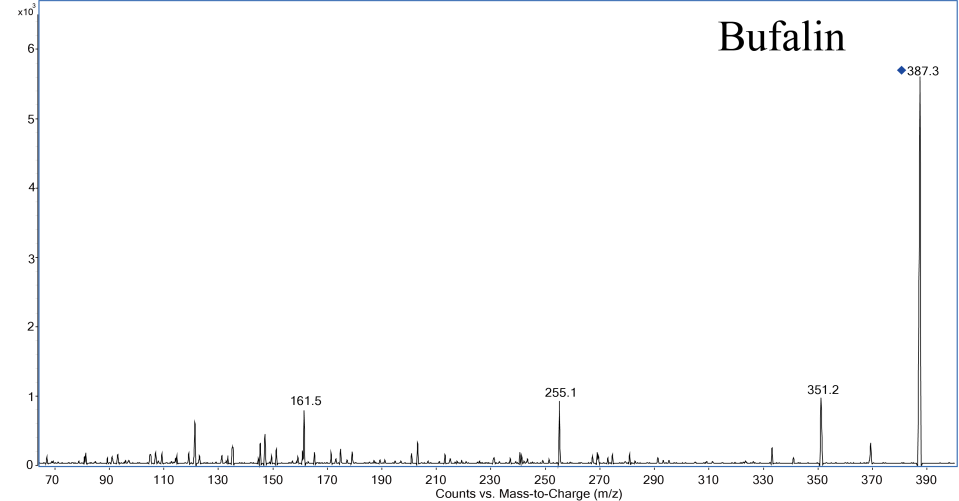


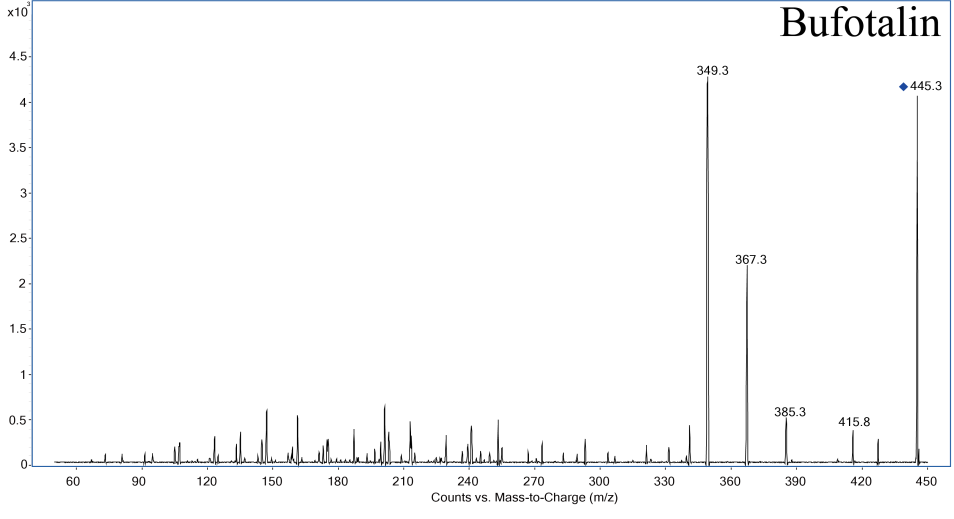


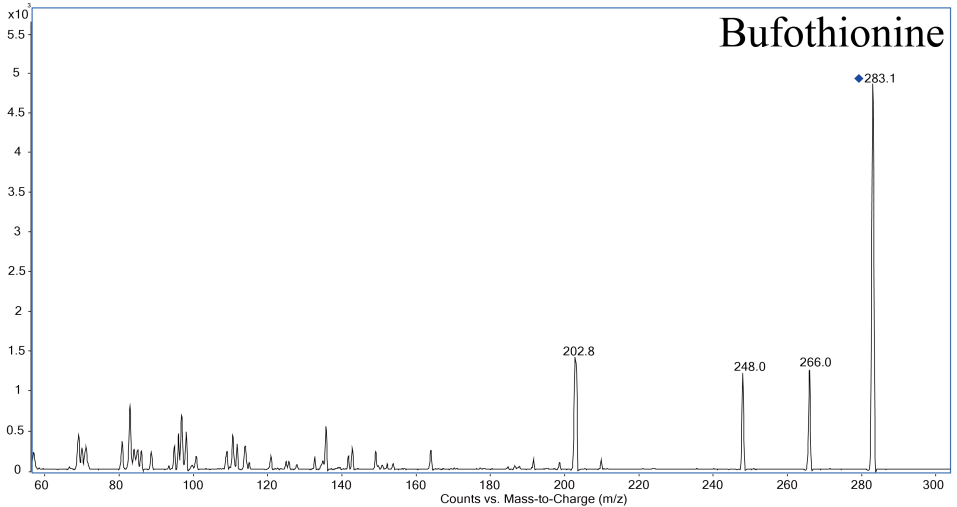


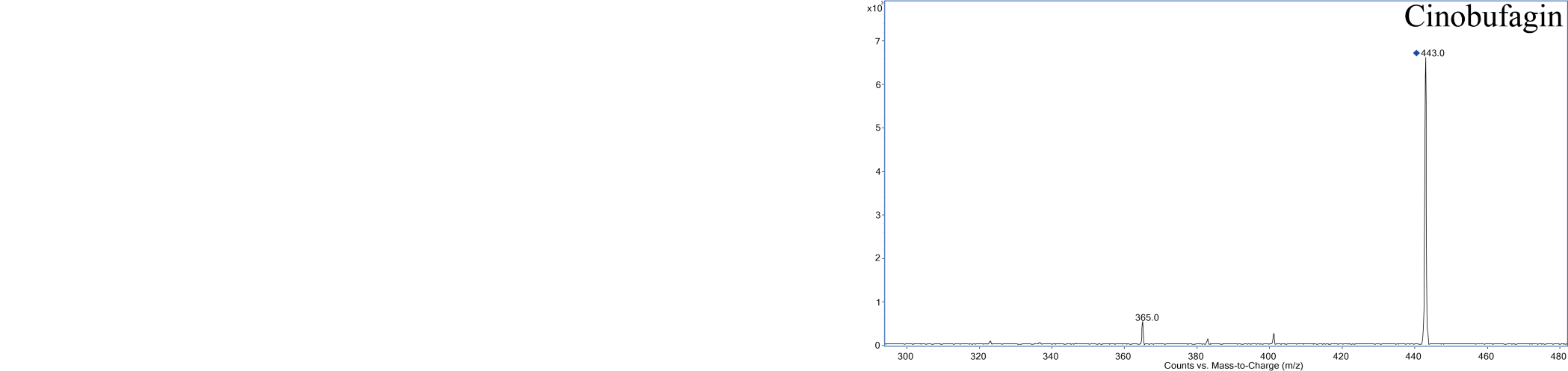


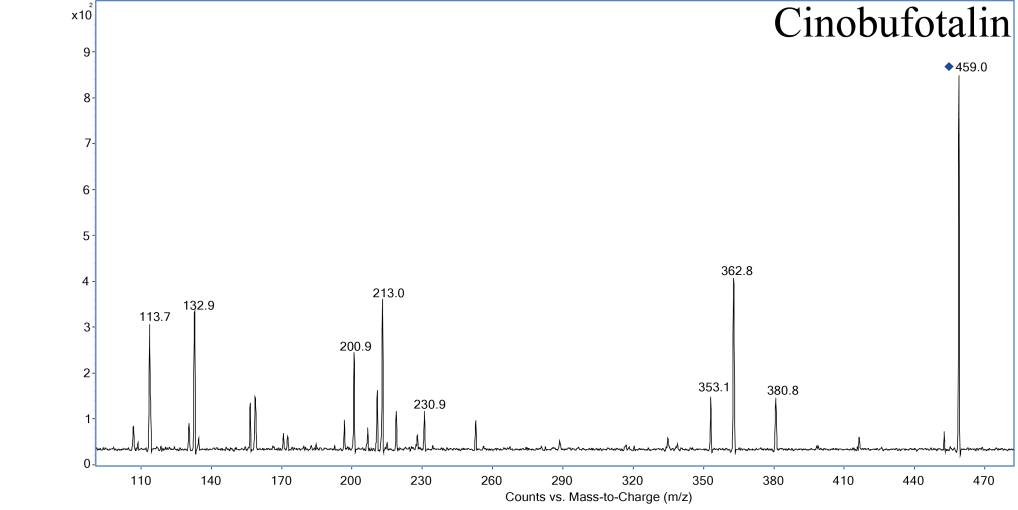


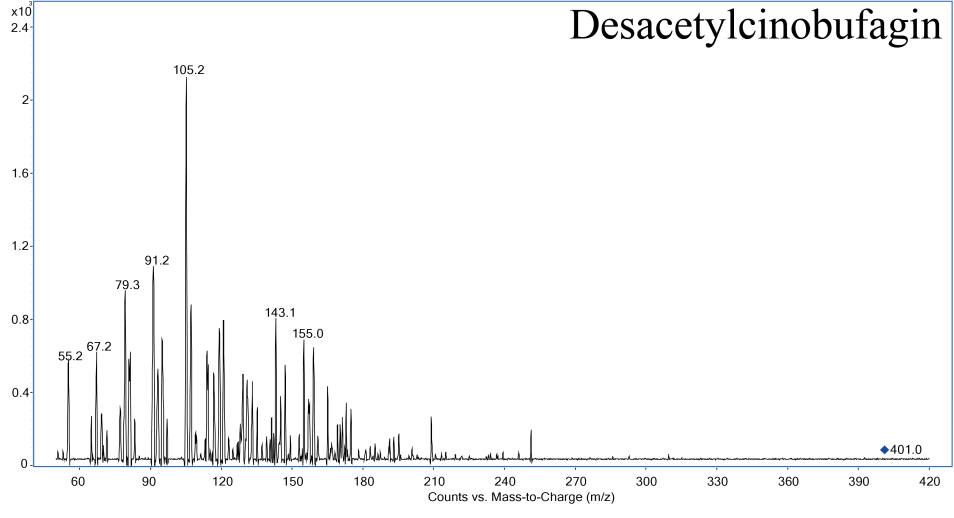


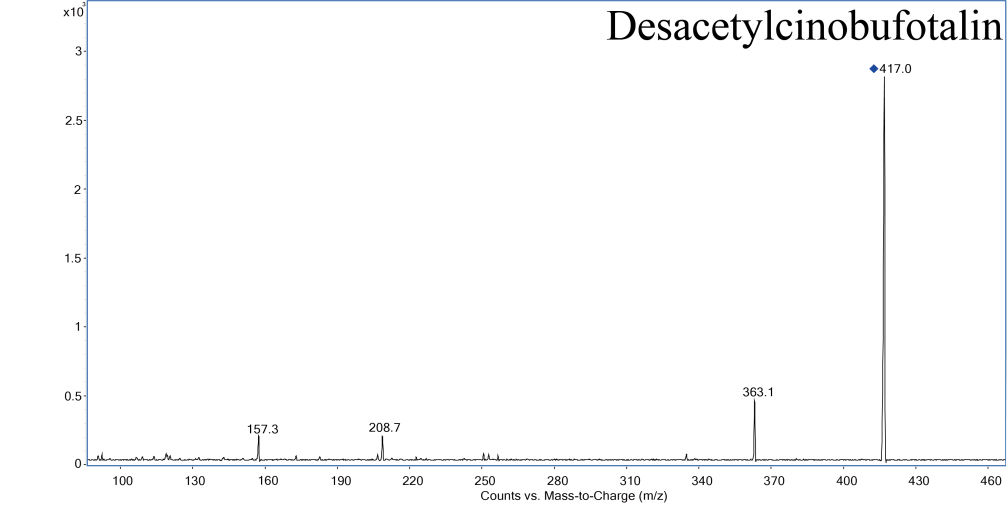


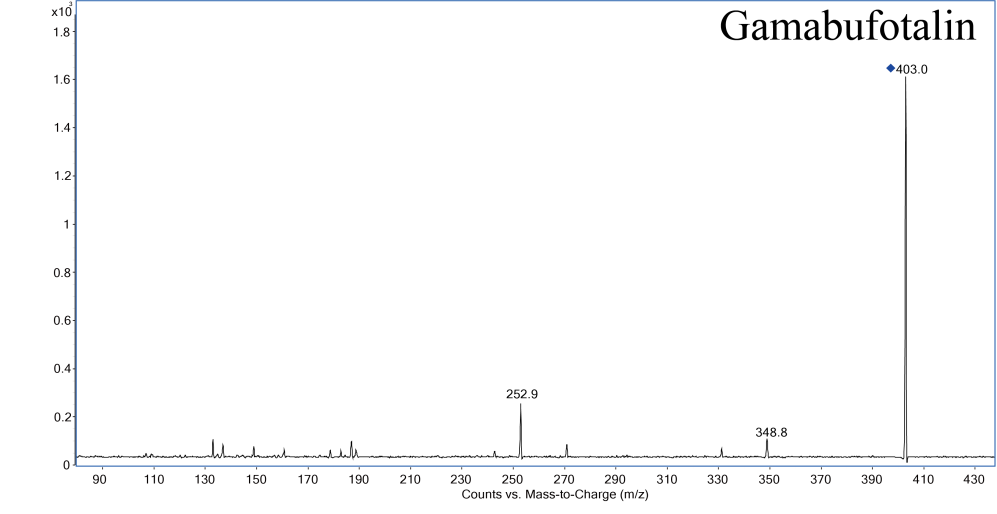


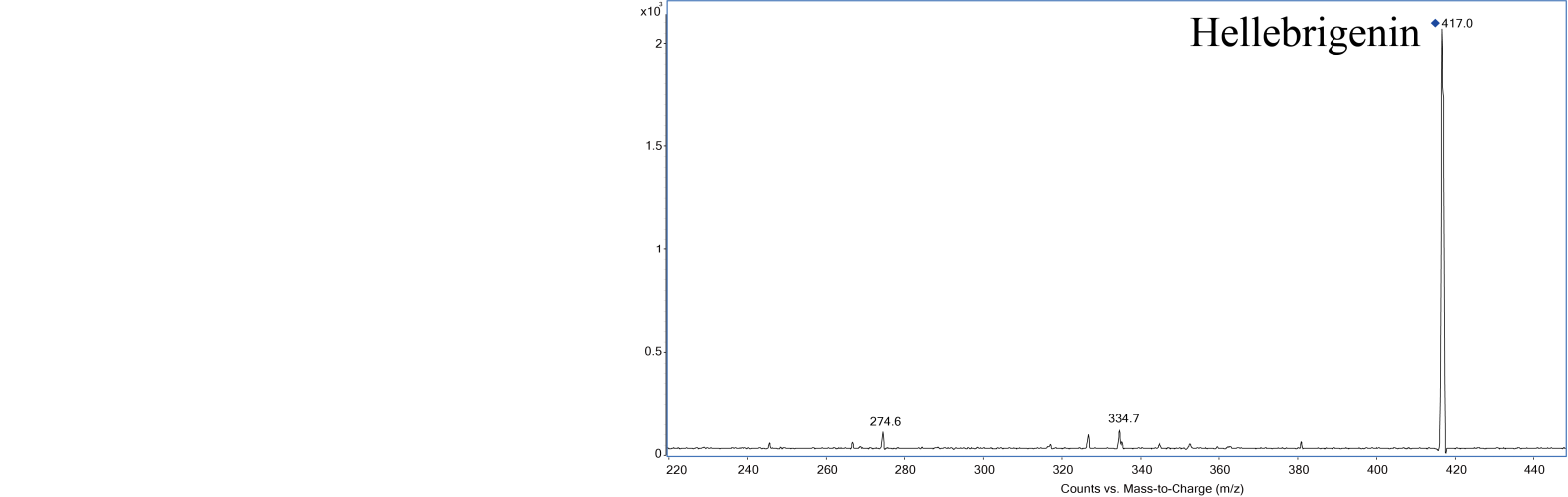


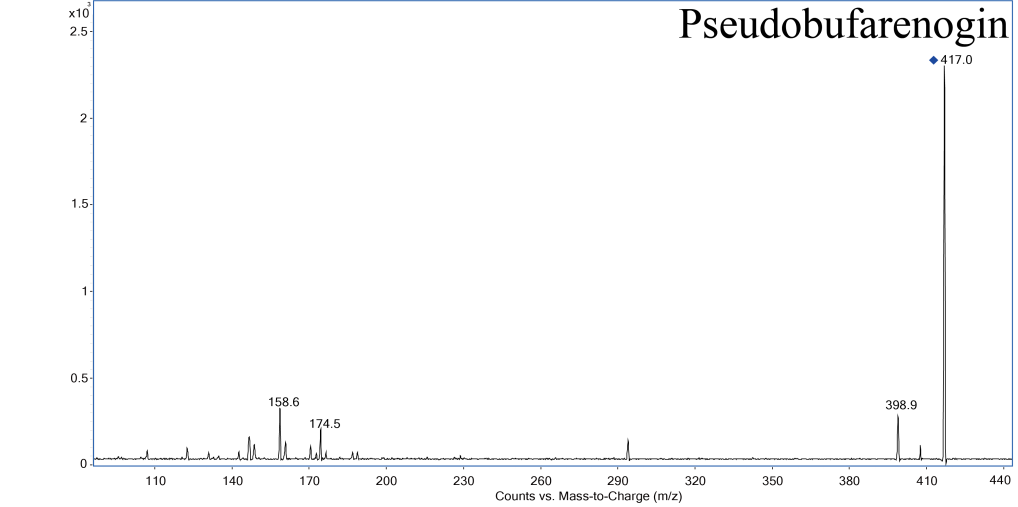


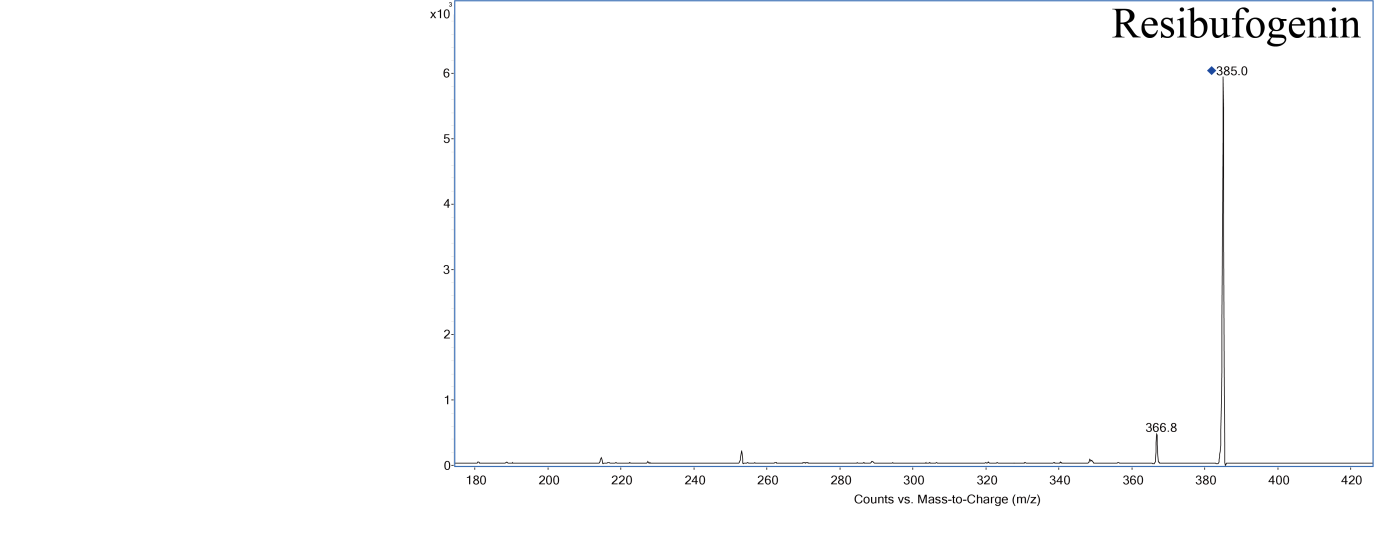


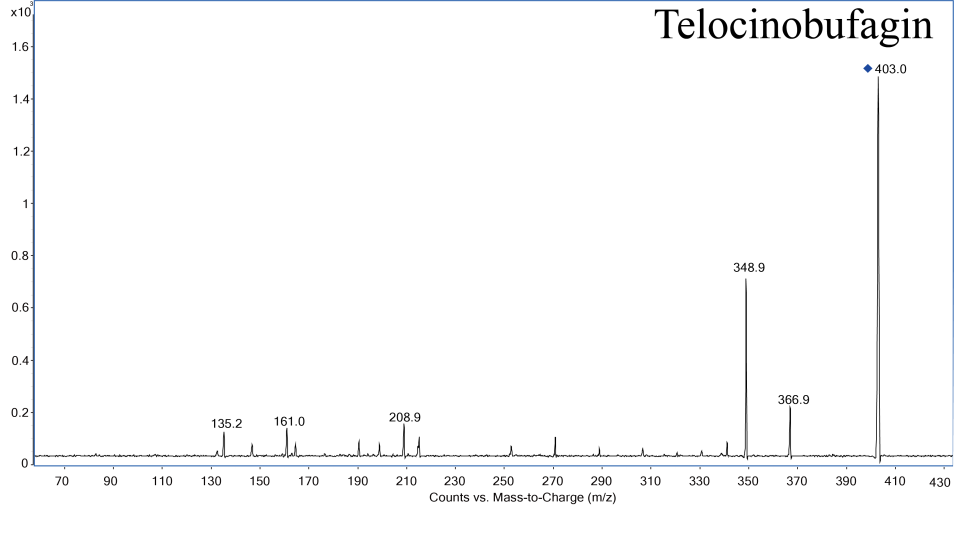


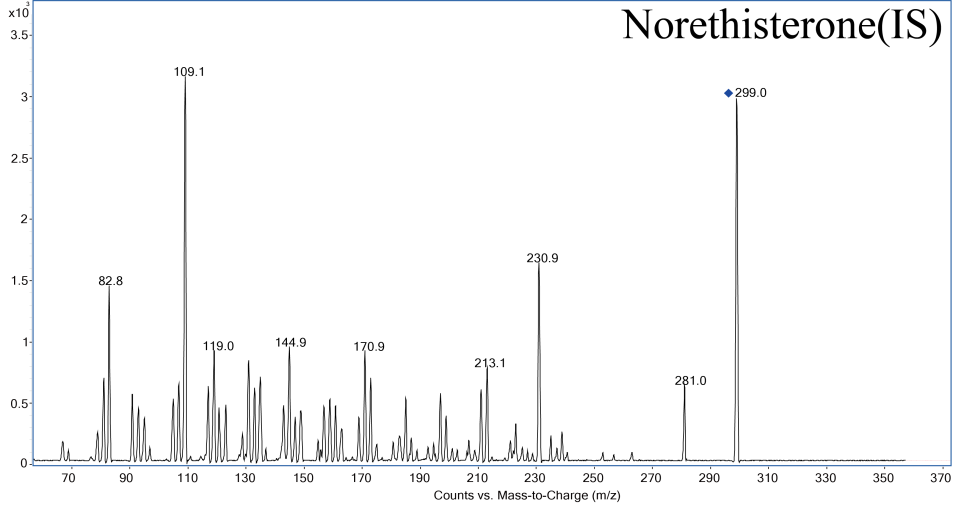


**Figure S1**  Product ion mass spectra of the analytes and IS.


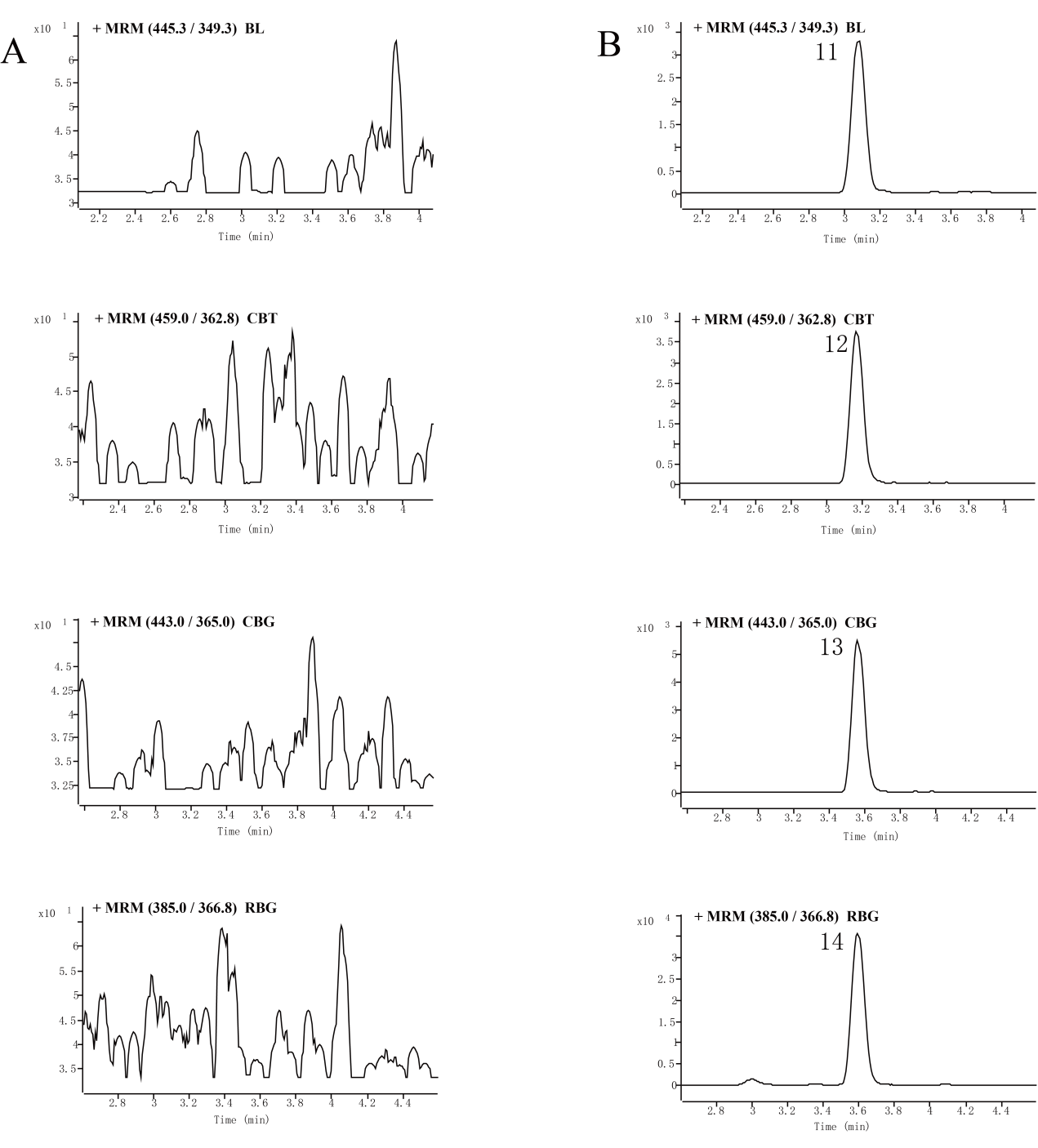


**Figure S2** The MRM chromatograms of four analytes were not detected in plasma. Blank rat plasma (A), blank rat plasma spiked with the analytes (B), Peak 11: bufotalin, 12: cinobufotalin, 13: cinobufagin, 14: resibufogenin.
